# S100A8 as a Promising Biomarker and Oncogenic Immune Protein in the Tumor Microenvironment: An Integrative Pancancer Analysis

**DOI:** 10.1155/2022/6947652

**Published:** 2022-03-18

**Authors:** Zixuan Wu, Dongli Jiang, Xuyan Huang, Minjie Cai, Kai Yuan, Peidong Huang

**Affiliations:** ^1^Guangzhou University of Chinese Medicine, Guangzhou, Guangdong Province, China 510006S; ^2^The Fifth Affiliated Hospital of Southern Medical University, Guangzhou, Guangdong Province, China 510009; ^3^Shantou Health School, Shantou, Guangdong Province, China 515061; ^4^Yunnan University of Chinese Medicine, Kunming, Yunnan Province, China 650500

## Abstract

**Background:**

S100 Calcium Binding Protein A8 (S100A8) is beneficial for cancer immunotherapy. However, the processes underlying its therapeutic potential have not been completely studied.

**Methods:**

The Cancer Genome Atlas provides raw data on 33 different cancer types. GEO made available GSE67501, GSE78220, and IMvigor210. We investigated S100A8's genetic changes, expression patterns, and survival studies. The linkages between S100A8 and TME, as well as its association with immunological processes/elements and the major histocompatibility complex, were explored to effectively understand the role of S100A8 in cancer immunotherapy. Three distinct immunotherapeutic cohorts were employed to examine the relationship between S100A8 and immunotherapeutic response.

**Results:**

S100A8 expression was high in tumor tissue. The overexpression of S100A8 is associated with poor clinical outcome in patients with overall survival. S100A8 is associated with immune cell infiltration, immunological modulators, and immunotherapeutic indicators. S100A8 overexpression is connected to immune-related pathways. However, no statistically significant connection between S100A8 and immunotherapeutic response was identified.

**Conclusions:**

S100A8 may be a reliable biomarker for tumor prognosis and a viable prospective therapeutic target for human cancer immunotherapy (e.g., GBM, KIRC, LGG, and LIHC).

## 1. Introduction

The S100 family consists of Ca^2+^ and Zn^2+^ binding proteins that are only present in invertebrates. S100A8 is a calcium-binding S100 protein with a low molecular weight. [1]. This compound is also known as calgranulin B or migration inhibitory factor-related protein 14MRP-14. S100A8 has been linked to tumor metastasis [2]. Protein levels in metastatic melanoma and prostate cancer increase [3, 4]. S100A8 overexpression is associated with tumorigenesis and poor differentiation in these tumors [5]. Although S100A8 has been studied in various cancers, its biological function in cancer remains contradictory and poorly understood.

Despite recent advances in anticancer therapies, such as chemoradiotherapy, targeted therapy, and immunotherapy, the overall survival rates for undetected, undiagnosed, or currently challenging to treat remain unsatisfactory [6, 7]. The tumorigenesis mechanisms should be urgently investigated, and new markers should be discovered for early detection, prognosis, and treatment. The tumor microenvironment (TME) consists of diverse cells and is crucial in the development, metastasis, and treatment resistance of human malignancies [8, 9]. However, the mechanism in which TME interacts with immune cells has not been determined. TME emerged recently as a new immunotherapy hotspot. Several therapeutic target blocking drugs have been used for cancer treatment since immunotherapy with immune checkpoint blockade and other strategies [10]. However, limited studies have focused on the significance of S100A8 and TME in cancer based on the current excellent design approach, and we aimed to use bioinformatics to explore SAS100A8 expression in 33 different malignant tumors and its potential effect on immune TME [11, 12].

**Figure 1:**
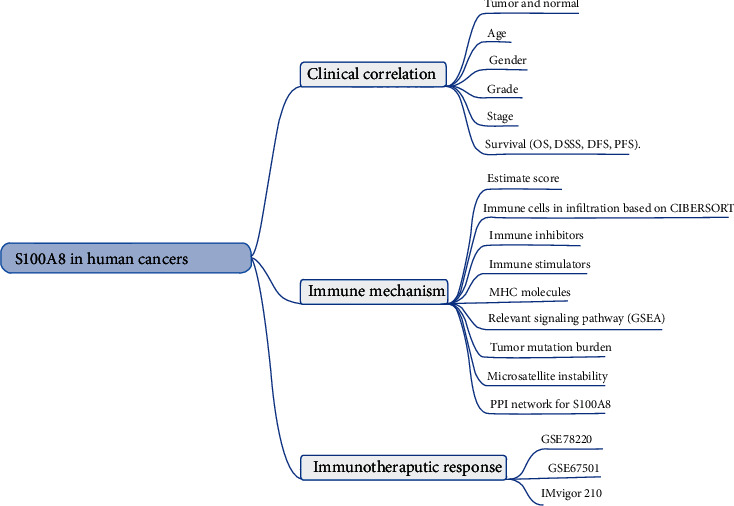
The analyses and indicators used in our study. Differential S100A8 expression analyses were performed in the clinical correlation section between different tissues, ages (=65 versus >65), genders, stages, and grades. The univariate Cox regression analysis was used for the survival correlation analyses. GSEA was used to investigate relevant signaling pathways based on S100A8 expression in the immune mechanism section. Wilcoxon test was performed on the S100A8 expression of nonresponder and responder groups in the immunotherapeutic response section.

This study was aimed at thoroughly examining S100A8 expression in 33 different malignant tumors and determining its potential effect on immune TME. Critical immunomodulators and dynamic immune biomarkers have been focused on. The strategy is shown in Figure 1.

## 2. Materials and Methods

### 2.1. Raw Data Collection and Processing

The Cancer Genome Atlas (TCGA) was used to collect 33 tumors gene expression patterns and clinical data [13]. The S100A8 status change was discovered using the cBioPortal database [13]. The genomic changes include copy number amplification, severe loss, an unknown missense mutation, and mRNA overexpression. We utilized log2 (TPM+1)-transformed expression data (tumor and matched normal tissue) to show the different analysis findings in parameter selection after extracting the S100A8 data by using Limma software.

### 2.2. Association of S100A8 with Survival and Clinical Stage

Overall survival (OS), disease-specific survival (DSS), disease-free survival (DFS), and progression-free survival (PFS) were used to assess S100A8's influence on cancer survival. We employed the log-rank and univariate Cox proportional hazards' models. Clinical factors such as age, gender, grade, and stage were considered for multivariate Cox regression. The stage survival plot module was used to evaluate the association between S100A8 expression and clinical stage.

### 2.3. Role of Immune Cell Infiltration and TME in S100A8 Expression

The link between S100A8 expression and tumor-infiltrating immune cells was examined and estimated. The stromalScore and immuneScore were computed after assessing the TME. Scatterplots were developed to assess tumor cell purity. The higher the expected ImmuneScore or StromalScore score, the higher the predicted ImmuneScore or StromalScore score.

Tumor mutation load (TMB) is a specific and accurate biomarker for predicting immunotherapy response. It can calculate the overall number of mutations per DNA megabase and identify alterations categorized as nucleotide insertions, base substitutions, or deletions [14]. Microsatellite instability (MSI) is a molecular tumor characteristic that is defined by spontaneous nucleotide loss or gain from short tandem repeat DNA sequences. To study the link between TMB and MSI, we employed the fmsb package.

### 2.4. Immunotherapeutic Response Analysis

This study, as previously noted, included and assessed three major independent immunotherapeutic cohorts, namely, GSE78220, GSE67501, and IMvigor210. The respondents include patients who achieved a complete or partial response rather than nonrespondents who had progressing disease or stable illness symptoms. The Wilcoxon test was then used to compare the levels of S100A8 expression in the respondent and nonrespondent groups.

### 2.5. Analysis of Gene Set Enrichment and Protein–Protein Interaction (PPI) Network

Gene set enrichment analysis (GSEA) was carried out in both the high and low-expression groups. Based on the KEGG and GO analyses, the top four words were displayed. Enrichment was significant in gene sets with ∣NES | >1, NOM *p* < 0.05, and FDR *q* < 0.05 [15]. In addition, we used the GeneMANIA web tool to create an S100A8 PPI network [16].

## 3. Results

### 3.1. Clinical Landscape of S100A8 Expression

**Figure 2:**
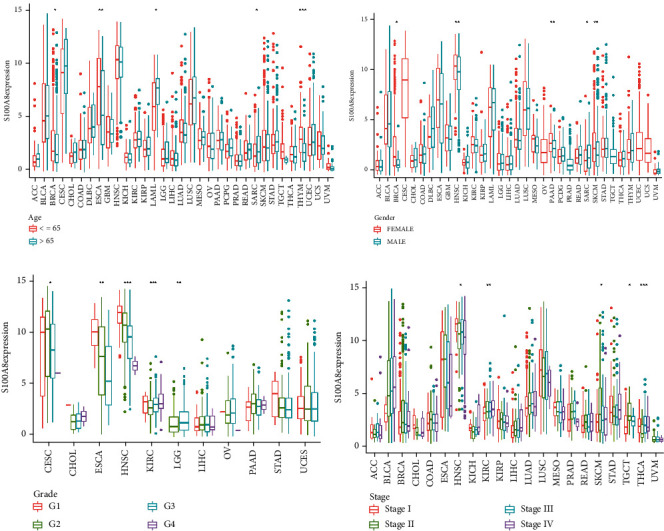
S100A8 has a clinical correlation. (a) Age. (b) Gender. (c) Grade. (d) Stage.

As demonstrated in Figure 2(a), S100A8 was differentially expressed in senior GBM patients but poorly expressed in BRCA, ESCA, LAML, SARC, and THYM. Significant gender variations were observed in the expression of BRCA, HNSC, PAAD, SARC, and SKCM (Figure 2(b)). S100A8 expression is associated with cancer stage in various malignancies, including CESC, ESCA, HNSC, KIRC, and LGG (Figure 2(c)). S100A8 expression is also associated with tumor stage in malignancies such as HNSC, KIRC, SKCM, TGCT, and THCA (Figure 2(d)).

**Figure 3:**
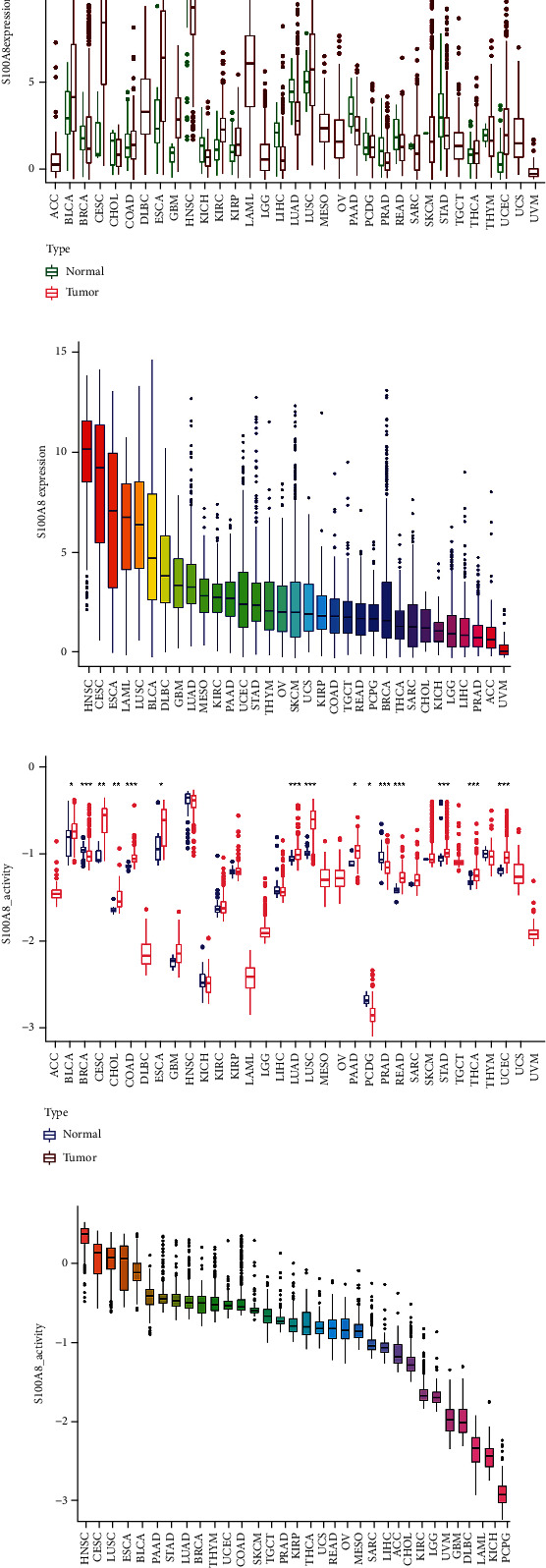
S100A8 activity generation and research. (a) Various S100A8 analyses. (b) S100A8 mean expression. (c) Different S100A8 activity analyses. (d) S100A8's mean activity.

As a sensitive indication, S100A8 may be an important new target or biomarker for cancer detection. The S100A8 expression was evaluated in tumors and normal tissues nearby to determine its relationship with cancer. S100A8 mRNA expression substantially differed in cancer samples from BRCA, CESC, ESCA, GBM, HNSC, KICH, KIRC, KIRP, LIHC, LUAD, PRAD, STAD, and UCEC, showing that S100A8 may operate as an oncogene in the advancement of several malignancies (Figure 3(a)). HNSC, CESC, ESCA, and LAML expression levels were high, as shown in Figure 3(b). S100A8 activity was considerably enhanced in tumor categories BLCA, CESC, CHOL, COAD, ESCA, LUAD, LUSC, PAAD, STAD, THCA, and UCEC but remarkably decreased in tumor categories BRCA, PCPG, PRAD, and READ, as shown in Figure 3(c).  Figure 3(d) demonstrates that the HNSC, CESC, LUSC, and ESCA are very active.

### 3.2. S100A8's Prognostic Value in Cancer

**Figure 4:**
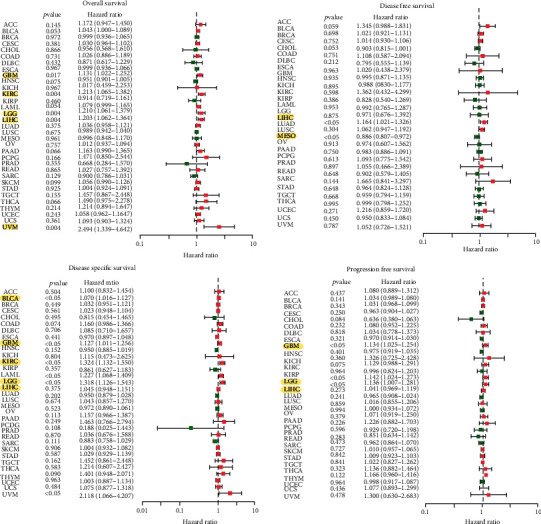
Univariate Cox regression analysis forest plots.

S100A8 expression was linked to OS in GBM, KIRC, LGG, LIHC, and UVM, according to the forest plots (Figure 4). S100A8 and DFS had a clear positive relationship in LIHC but a negative relationship in LUSC. S100A8 is a risk factor for BLCA, GBM, KIRC, LGG, LIHC, and UVM in the DSS. The PFS forest plot confirmed the risk effect of S100A8 expression in GBM, LGG, and LIHC. The plot allowed the researchers to discover additional malignancies, where S100A8 expression was deemed to be a risk factor, such as LIHC and UVM. S100A8 expression was highly correlated with survival in several malignancies, although it was not directly connected to clinical characteristics (e.g., GBM, KIRC, LGG, and LIHC).

### 3.3. S100A8 Expression and Immune Infiltrating Levels in Cancer

**Figure 5:**
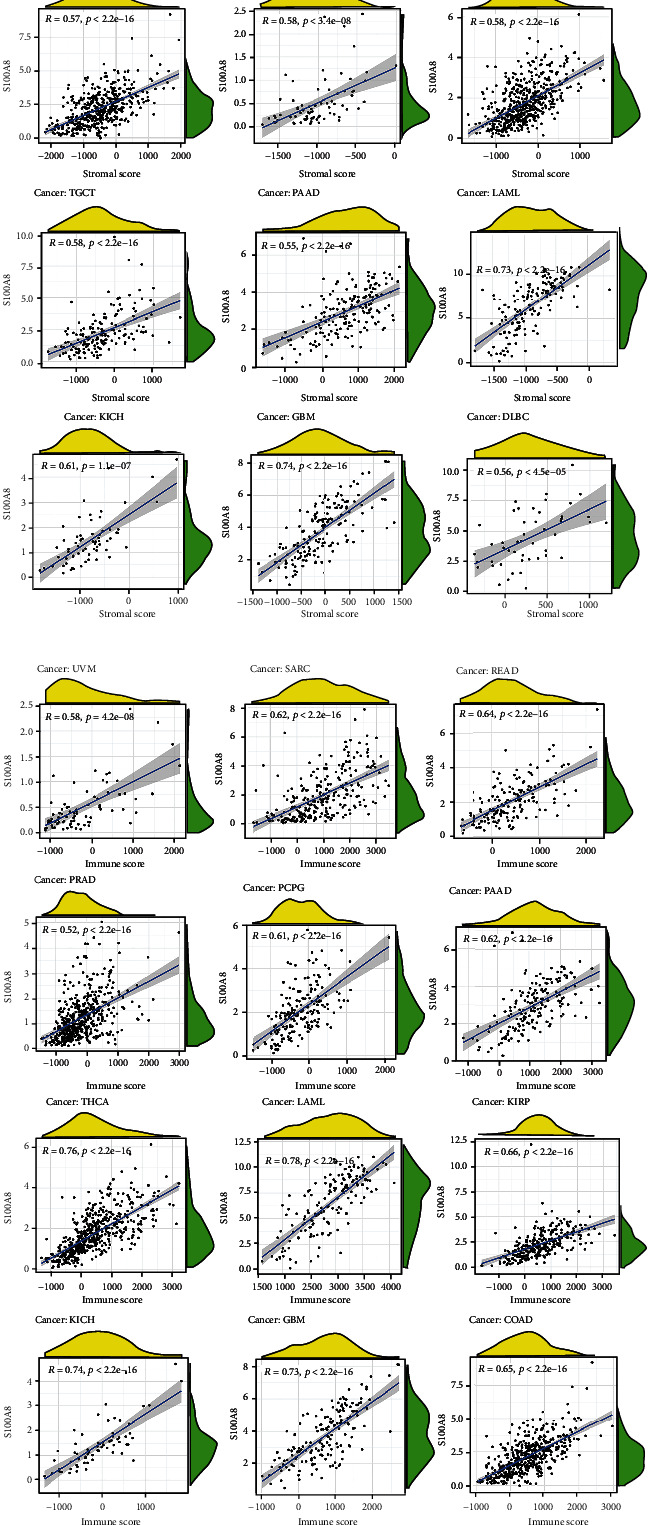
S100A8 expression and the ESTIMATE score. (a) StromalScore. (b) .ImmuneScore.

**Figure 6:**
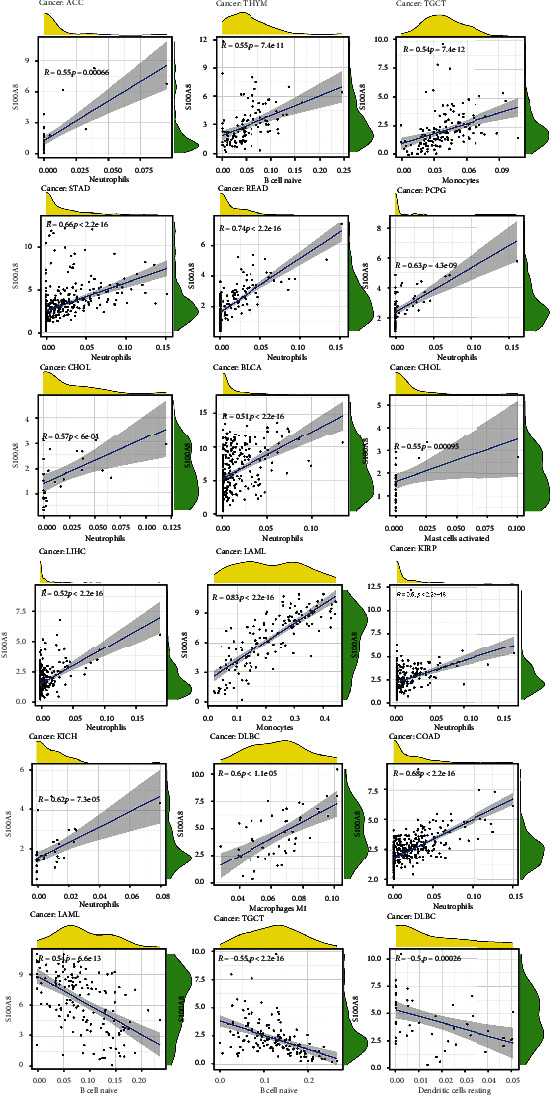
The amount of immune infiltration in malignancies is correlated with S100A8 expression.

We calculated the coefficient of S100A8 expression and immune infiltration level to determine their relationship with the degree of immune infiltration in diverse cancers.  Figure 5 summarizes the stromal and immunological scores. S100A8 expression was linked to the immunological scores COAD, DLBC, GBM, KICH, LAML, PAAD, TGCT, THCA, and UVM, as well as the stromal scores COAD, DLBC, GBM, KICH, KIRP, LAML, PAAD, PCPG, PRAD, READ, SARC, THCA, and UVM. The S100A8 expression was negatively related to mast cells activated in CHOL, dendritic cells resting in DLBC, macrophages M1 in DLBC, monocytes in LAML and TGCT, B cells naïve in THYM, TGCT, and LAML, and neutrophils in READ, CHOL, COAD, KICH, KIRP, ACC, LIHC, PCPG, BLCA, and STAD (Figure 6).

### 3.4. Analysis of S100A8 Expression and Immune Modulators

**Figure 7:**
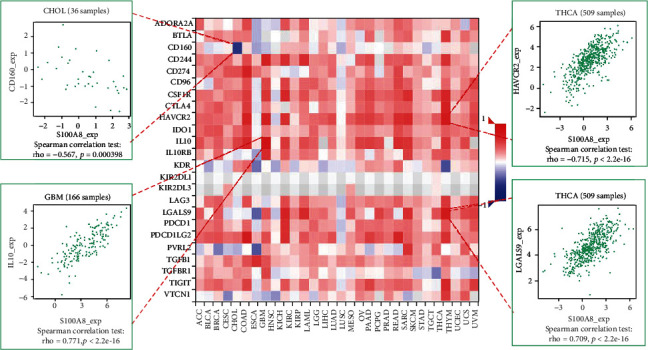
S100A8 expression and immune inhibitors.

**Figure 8:**
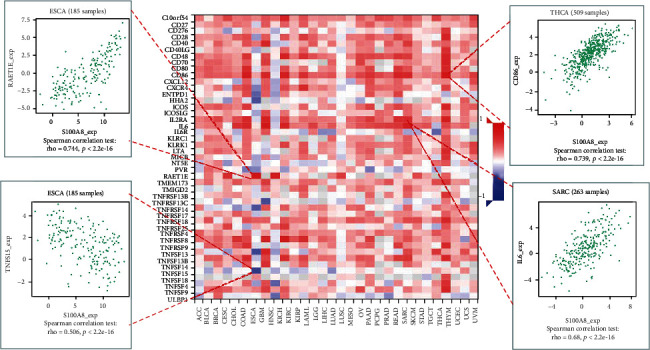
S100A8 and immune stimulators.

**Figure 9:**
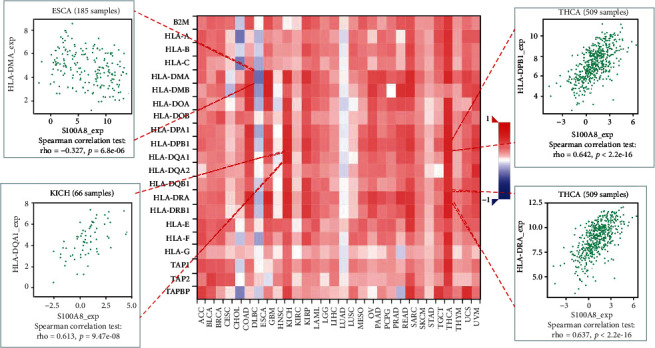
S100A8 and MHC molecules.

The relationship between S100A8 expression and immune modulators was also studied.  Figure 7 displays the findings of a research that included 24 different types of immune inhibitors. S100A8 was linked to IL10 in GBM, HAVCR2, and LGALS9 in THCA but negatively associated CD160 in CHOL. Based on correlation analyses, S100A8 expression was favorably linked with RAET1E in ESCA, CD86 in THCA, and IL6 in SARC, but negatively associated with TNFSF15 in ESCA (Figure 8). Furthermore, as shown in Figure 9, S100A8 expression was related to HLA-DQA1 in KICH and HLA-DPB1 and HLA-DRA in THCA, but not with HIL-DMA in ESCA.

### 3.5. Immunotherapeutic Markers and S100A8 Response

**Figure 10:**
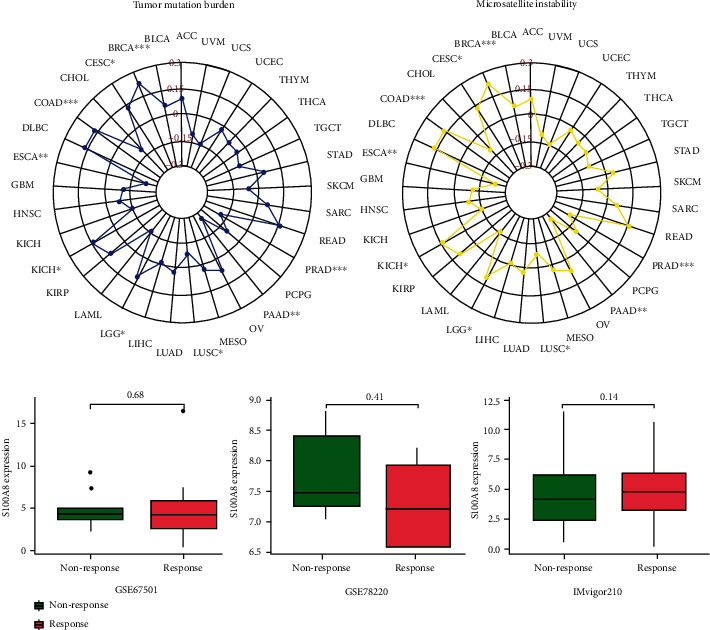
Relationship between S100A8 and immunotherapeutic indicators and response.

The relationship between S100A8 and two novel dynamic indicators of immune checkpoint blockage (TMB and MSI) was further investigated. S100A8 expression is linked to TMB in LGG, CESC, PRAD, BRCA, PAAD, ESCA, COAD, LUSC, and KIRC, as shown in Figure 10. MSI was associated with a positive relationship in LGG, CESC, COAD, KIRC, and BRCA and a negative relationship in ESCA, LUSC, PAAD, and PRAD. In the three cohorts, no significant difference was observed in the S100A8 expression between the respondent and nonrespondent groups, as shown in Figure 10. Patients with reduced S100A8 expression were more sensitive to immunotherapy in the analyzed cohorts.

### 3.6. S100A8 PPI Network in Cancers and GSEA

**Figure 11:**
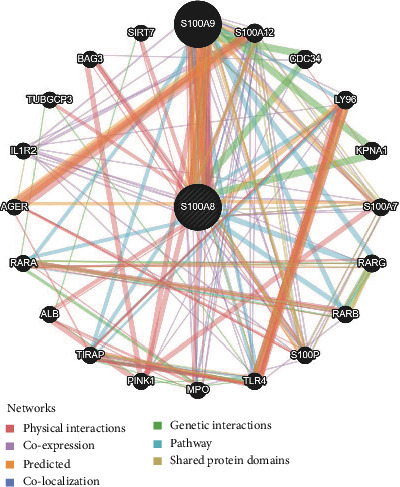
PPI network of S100A8.

**Figure 12:**
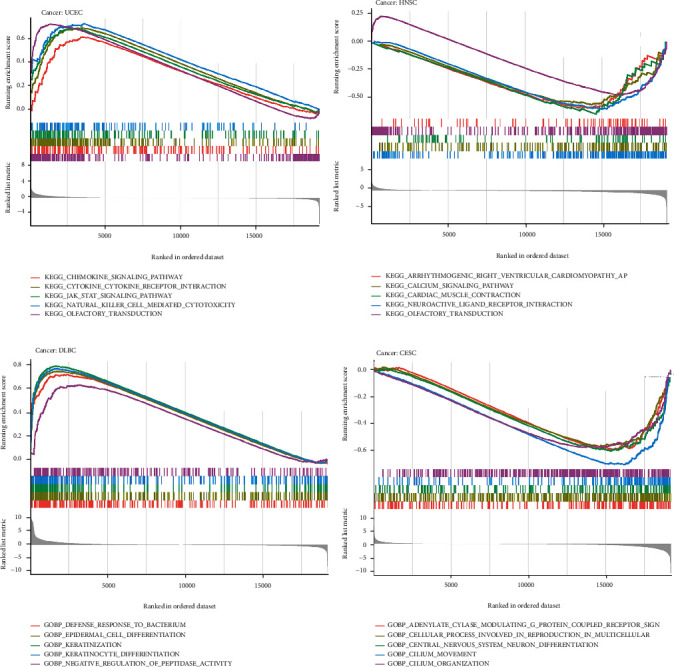
GSEA for samples with S100A8 expression. (a) The low expression. (b) The high expression sample. (c) The low expression. (d) The high expression. (a + b): The enriched gene sets in KEGG. (c + d): The enriched gene sets in GO.

To explore the underlying processes by which S100A8 contributes to cancer carcinogenesis, we constructed an S100A8 PPI network (Figure 10). S100A8 made solid physical contact with S100A9, as shown in Figure 11, and this condition is essential for cancer metastasis. S100A8 and S100A9 are involved in inflammatory responses, including cell survival; they induced EoL-1 cell migration via PKC, AKT, and MAPK phosphorylation and NF-*κ*B translocation [17]. S100A8 and S100A9, which are endogenous risk-associated molecular models that recognize Toll-like receptor 4 and the receptor for advanced glycation end products, respectively, influence atherosclerosis development and progression by altering endothelial permeability and promoting intraplaque inflammation [18]. Furthermore, S100A8 could be linked to S100A12 and CD34. The functional enrichment of high and low S100A8 expression was then determined using GSEA (Figure 12). High S100A8 expression was connected with metabolic-related activities, such as arrhythmogenic right ventricular cardiomyopathy arvc, calcium signaling pathway, cardiac muscle contraction, neuroactive ligand receptor interaction, and olfactory transduction, according to the KEGG enrichment term. According to the GO enrichment term, high S100A8 expression is primarily associated with the adenylate cyclase modulating g protein coupled receptor signaling pathway, cellular process involved in multicellular organism reproduction, central nervous system neuron differentiation, cilium movement, and cilium organization.

## 4. Discussion

Inflammation is the primary protective mechanism of the body. During inflammation, many immunocytes and chemicals establish a massive regulatory network, removing endogenous and foreign harmful substances to protect the body. However, network imbalances, such as excessive inflammatory responses and extended inflammatory state, may aggravate tissue damage [19, 20]. S100A8 and S100A9 are members of the S100 calcium-binding protein family, and when these proteins combine (S100A8/A9), they create the physiologically relevant version known as calprotectin. S100A8/A9 is granulocyte- and monocyte-specific and is involved in various pathological processes, such as inflammation, infection, and autoimmune diseases [21, 22]. S100A8/A9 proinflammatory action involves leukocyte recruitment, cytokine and chemokine secretion enhancement, and leukocyte adhesion and migration modulation [23]. S100A8/A9 proteins play a prominent role in inflammation, immunological responses, and infectious illnesses [24, 25]. Furthermore, S100A8/A9 performs two distinct but linked activities in intracellular and extracellular microenvironments. However, the effect of S100A8/A9 on TME invasion in various malignancies remains unclear. Considering the relevance of S100A8/A9 in the physiology of inflammation, it is a viable candidate as a diagnostic biomarker and therapeutic target for inflammation-related illnesses, and its clinical potential needs further exploration.

Contrary to common belief, S100A8 is a toxicant-related transcription factor that is required for immunological TME and may have immunotherapeutic potential. Therefore, further research should focus on S100A8, particularly TME, immune cells, immunological modulators, and the immunotherapeutic response. The researchers aimed to understand the pathways that may connect S100A8 to immune-related variables in pancancer. First, the association between S100A8 and clinical factors was explored, and no significant changes in age, gender, or tumor stage were found in the majority of cancer types, supporting prior findings. S100A8 expression has only a marginal predictive effect in several malignancies, including breast cancer [26]. S100A8 has also been discovered as a protooncogene in several malignancies, including lung cancer [27], colorectal cancer [28], and cholangiocarcinoma [29]. The proteins S100A9 and S100A8 are involved in a paracrine feedback loop between pancreatic cancer cells and monocytes [30]. Tumor-infiltrating monocytes/macrophages increase tumor invasion and migration by increasing the expression of S100A8 and S100A9 in cancer cells [31]. We anticipate that the therapeutic modulation of S100A8 activity in many tumor types might be a realistic clinical approach based on the results obtained.

Furthermore, when the transcriptional level was compared to the S100A8 activity score, it partially matched the total S100A8 activation in several tumors (e.g., BRCA, CESC, ESCA, LUAD, PRAD, STAD, and UCEC). Therefore, S100A8 activation was expressed at the transcriptional level in these malignancies. In certain malignancies, S100A8 expression and activity were incompatible (e.g., GBM, HNSC, KICH, KIRC, KIRP, and LIHC), possibly because posttranscriptional protein modification or protein metabolism influenced S100A8 expression. In GBM, KIRC, LGG, LIHC, and UVM, the S100A8 expression was associated with OS. De Ponti et al. revealed that S100A8 is essential for the evolution of noninflammatory liver tumors, and it might be a therapeutic target for the treatment of LIHC produced in noncirrhotic liver [32]. Gielen et al. reported a tumor-grade-dependent increase in myeloid-derived suppressor cells (MDSCs) in glioma patients' blood based on the assessment of the presence and activation status of MDSCs in glioma patients' blood and tumor by measuring S100A8/9 and arginase levels [33]. Both S100A8/A9 and CA15-3 serum levels were considerably high in patients with breast cancer, and it was positively linked with tumor size, showing that the S100A8/A9 heterodimer might be regarded a possible biomarker for BRCA diagnosis and prognosis [34]. KIRC transcriptome profiles with considerably greater levels of S100A8 expression were implicated in KIRC progression. S100A8 has increased in prominence as a target for anticancer treatment research because of its essential function in triggering inflammatory pathways that aid in cancer spread [35]. These studies support the validity and plausibility of our findings, and S100A8 may be associated with the oncology process in these cancer patients (e.g., GBM, KIRC, BRCA, and LIHC), implying that S100A8 is an oncogene.

To assess S100A8's potential utility, we further investigated the relationship between S100A8 and immune-cell infiltration. S100A8 and neutrophils have been linked with BLCA, KIRP, CHOL, COAD, STAD, LIHC, ACC, PCPG, READ, and KICH. Moreover, S100A8 modulates tumor development and immunological responses in TME-associated macrophages [36]. S100A8 may be involved in neutrophil activation and the subsequent activation of an immunosuppressive response [37]. In immunoinhibitor, CD160 has the most significant unfavorable association with S100A8. Except for ESCA, the majority of immune stimulants and MHC molecules have a favorable association with S100A8; based on this finding, a unique regulatory mechanism in ESCA immunotherapy can be identified. GSEA further demonstrated that increased S100A8 expression was mostly associated with metabolic-related activities. The dysregulation of cytokine and adipocytokine expression in adipose tissue characterizes metabolic inflammation [38]. S100A8 is a protein that binds calcium and zinc and modulates inflammatory and immunological responses [39]. It can activate ITGAM/ITGB and TLR4 and a signaling pathway involving MEK-ER to enhance cytokine and chemokine production and to control leukocyte adhesion and migration [40]. Extracellular proinflammatory activities include leukocyte recruitment, stimulation of cytokine and chemokine production, and leukocyte adhesion and migration regulation [41]. According to the current findings, increased S100A8 expression may alter innate immunity in certain malignancies by activating metabolic-related pathways.

Furthermore, two immunotherapeutic biomarkers, namely, TMB and MSI, have a strong connection with S100A8 in diverse malignancies. In general, the TMB accurately predicts the tumor-neoantigen load. The greater the number of somatic mutations in a tumor, the more probable it is to develop neoantigens [42]. MSI is a strong mutator phenotype induced by poor DNA mismatch repair that may serve as a prognostic signal for immunotherapy [43]. S100A8 is inversely related to TMB and MSI in ESCA, LUSC, PAAD, and PRAD. However, it was linked to both biomarkers in BRCA, CESC, KIRC, and LGG. This finding demonstrates that S100A8 might indirectly influence the immune response to past malignancies. S100A8 and immunotherapeutic response were investigated, but no significant differences were found in any of the cohorts evaluated. This study provided more information about S100A8's latent involvement in tumor immunology and its potential application as a cancer biomarker. Meanwhile, considering that our immunotherapeutic response research only included three relevant cohorts, S100A8's specific immunotherapeutic response was not determined. In the future, more important immunotherapeutic populations should be explored.

This study provided more information about the role of S100A8 in cancer immunotherapy. It reveals a relationship between S100A8 and critical immunological markers, which might help researchers better understand the potential linkages between S100A8 and the immune system. The present study has some limitations. The results provide a foundation for theoretical foundations and analytical concepts. We only built a verified S100A8 prediction signature by using the TCGA datasets and were unable to gather enough external data from other publicly available sources to verify the model's credibility. Furthermore, the bioinformatics research revealed some interesting details concerning S100A8's function in cancer. Biological research, both *in vitro* and *in vivo*, is required to confirm our results and improve treatment effectiveness.

## 5. Conclusions

In conclusion, our findings revealed a close relationship and prognostic significance of S100A8 expression in various human cancers. S100A8 can be a novel cancer treatment target. Our findings also provide insight into S100A8's important involvement in carcinogenesis and metastasis and a proposed mechanism through which S100A8 expression modulates tumor immunology and metabolic activity. Our findings can contribute to the identification of a relationship between S100A8 expression and immunological TME to further elucidate their possible function in cancer genesis and progression and thus provide immuno-based anticancer therapy.

## Data Availability

Patients who granted informed consent to use their data have been uploaded to publicly accessible TCGA databases. At their leisure, users can get and publish relevant articles depending on the needed data. Our study does not involve ethical problems or conflicts of interest, because it is based on open-source data. The [diseases] data used to support the findings of this study have been deposited into the [TCGA] repository ((https://portal.gdc.cancer.gov/).

## Abbreviation

S100A8: S100 Calcium Binding Protein A8
TCGA: The Cancer Genome Atlas
GO: Gene Ontology
GEO: Gene Expression Omnibus
KEGG: Kyoto Encyclopedia of Genes and Genomes
GSEA: Gene set enrichment analysis
TME: Tumor microenvironment
MSI: Microsatellite instability
TMB: Tumor mutational burden
OS: Overall survival
DSS: Disease-specific survival
CI: Confidence intervals
HR: Hazard ratios
TIICs: Tumor-infiltrating immune cells
PPI: Protein-protein interaction
PFS: Progression-free survival
CR: Complete response
PR: Partial response
PD: Progressing disease
SD: Stable illness.
